# Designed biosynthesis of 25-methyl and 25-ethyl ivermectin with enhanced insecticidal activity by domain swap of avermectin polyketide synthase

**DOI:** 10.1186/s12934-015-0337-y

**Published:** 2015-09-24

**Authors:** Ji Zhang, Yi-Jun Yan, Jing An, Sheng-Xiong Huang, Xiang-Jing Wang, Wen-Sheng Xiang

**Affiliations:** School of Life Science, Northeast Agricultural University, No. 59 Mucai Street, Xiangfang District, Harbin, 150030 China; State Key Laboratory for Biology of Plant Diseases and Insect Pests, Institute of Plant Protection, Chinese Academy of Agricultural Sciences, No. 2 Yuanmingyuan West Road, Haidian District, Beijing, 100193 China; Kunming Institute of Botany, Chinese Academy of Sciences, No. 132 Lanhei Road, Panlong District, Kunming, 650201 China

**Keywords:** 25-Ethyl ivermectin, 25-Methyl ivermectin, Domain swap, Insecticidal activity

## Abstract

**Background:**

Avermectin and milbemycin are important 16-membered macrolides that have been widely used as pesticides in agriculture. However, the wide use of these pesticides inevitably causes serious drug resistance, it is therefore imperative to develop new avermectin and milbemycin analogs. The biosynthetic gene clusters of avermectin and milbemycin have been identified and the biosynthetic pathways have been elucidated. Combinatorial biosynthesis by domain swap provides an efficient strategy to generate chemical diversity according to the module polyketide synthase (PKS) assembly line.

**Results:**

The substitution of *ave*DH2-KR2 located in avermectin biosynthetic gene cluster in the industrial avermectin-producing strain *Streptomyces avermitilis* NA-108 with the DNA regions *mil*DH2-ER2-KR2 located in milbemycin biosynthetic gene cluster in *Streptomyces bingchenggensis* led to *S. avermitilis* AVE-T27, which produced ivermectin B1a with high yield of 3450 ± 65 μg/ml. The subsequent replacement of *ave*LAT-ACP encoding the loading module of avermectin PKS with *mil*LAT-ACP encoding the loading module of milbemycin PKS led to strain *S. avermitilis* AVE-H39, which produced two new avermectin derivatives 25-ethyl and 25-methyl ivermectin (**1** and **2**) with yields of 951 ± 46 and 2093 ± 61 μg/ml, respectively. Compared to commercial insecticide ivermectin, the mixture of 25-methyl and 25-ethyl ivermectin (**2**:**1** = 3:7) exhibited 4.6-fold increase in insecticidal activity against *Caenorhabditis elegans*. Moreover, the insecticidal activity of the mixture of 25-methyl and 25-ethyl ivermectin was 2.5-fold and 5.7-fold higher than that of milbemycin A3/A4 against *C. elegans* and the second-instar larva of *Mythimna separate*, respectively.

**Conclusions:**

Two new avermectin derivatives 25-methyl and 25-ethyl ivermectin were generated by the domain swap of avermectin PKS. The enhanced insecticidal activity of 25-methyl and 25-ethyl ivermectin implied the potential use as insecticide in agriculture. Furthermore, the high yield and genetic stability of the engineered strains *S. avermitilis* AVE-T27 and AVE-H39 suggested the enormous potential in industrial production of the commercial insecticide ivermectin and 25-methyl/25-ethyl ivermectins, respectively.

**Electronic supplementary material:**

The online version of this article (doi:10.1186/s12934-015-0337-y) contains supplementary material, which is available to authorized users.

## Background

**Fig. 1:**
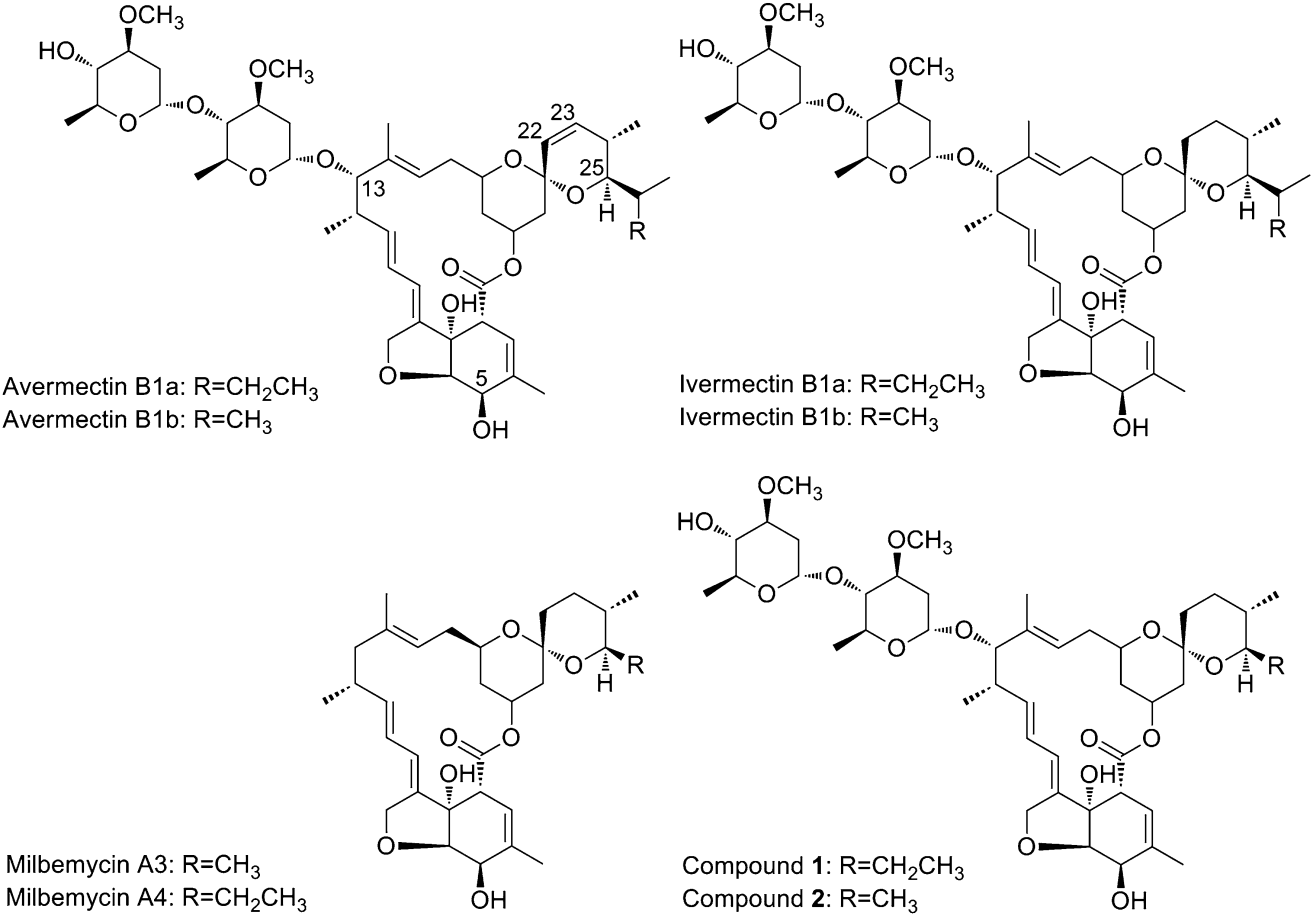
The structures of avermectins, milbemycins, and 25-methyl/25-ethyl ivermectin (**1** and **2**)

Avermectins and milbemycins (Fig. 1), the 16-membered macrolide antibiotics with potent anthelmintic and insecticidal activity, have been widely used for broad-spectrum parasite control in agricultural, medical, and veterinary fields [1–3]. They are structurally related compounds with structural differences at C25, C22–C23, and C13, leading to their own unique ‘spectral fingerprint’ with various strengths and dosage-limiting species. The subsequent chemically modification of avermectins and milbemycins resulted in series of analogs, some of which are commercially developed as anthelmintics and insecticides, such as ivermectin, selamectin, abamectin, emamectin, doramectin, milbemycin oxime, lepimectin, and latidectin [2]. Among these analogs, ivermectin (22,23-dihydroavermectin B1), showing the same effective antiparasitic activity and lesser toxic side effect than avermectins B1, has been worldwide used as an anthelmintic for livestock and companion animals and as an agricultural insecticide. Moreover, ivermectin has also been applied in human medicine, particularly treatment of onchocerciasis and lymphatic filariasis [4]. In the case of milbemycins, moxidectin is currently undergoing a phase III clinical trial to compare its efficacy with ivermectin in subjects with *Onchocerca volvulus* infection [1]; milbemycin oxime has been used against intestinal nematodes in dogs and cats, against adult heartworm in dogs, and against ectoparasites in companion animals [5]. Recently, it has been reported that ivermectin, selamectin and moxidectin demonstrated antibacterial activity against *Mycobacterium tuberculosis*, especially the multidrug-resistant and extensively drug-resistant clinical strains [1]. The approval for clinical and veterinary uses as well as the documented pharmacokinetic and safety profiles of these compounds make them potential therapeutic options for treating *M. tuberculosis*. The outstanding activities of avermectins and milbemycins together with the potential uses in the filed of human medicine and agriculture stimulate the semisynthetic derivatives of avermectins and milbemycins. On the other hand, the wide use of avermectins in agriculture has inevitably caused serious drug resistance [2], it is therefore imperative to develop novel avermectins.

The previously established industrial process for preparing the analogs of avermectins and milbemycins involves extracting avermectins or milbemycins from the fermentation broth and the subsequently chemical modification. However, this process suffers several drawbacks, such as the expensive cost, the heavy metal pollution, and low efficiency [3, 6–8]. Fortunately, the combinatorial biosynthesis and genetic manipulation provide alternative and efficient strategies to generate the known semi-biosynthetic polyketides and new hybrid compounds according to the module polyketide synthase (PKS) assembly line [3, 9, 10]. For example, on the basis of understanding the biosynthetic mechanism of avermectin, 22,23-dihydroavermectins including ivermectin were successfully produced by direct fermentation of engineered *S. avermitilis*, in which the DNA region encoding the dehydratase (DH) and ketoreductase (KR) domains of module 2 from the avermectin PKS was replaced by the DNA fragment encoding the DH, enoylreductase (ER), and KR domains of module 13 from the rapamycin PKS, module 4 from the pikromycin PKS, or module 3 from the oligomycin PKS [6, 8, 11]. Traditionally, doramectin is produced by a *bkd* mutant *S. avermitilis*, which blocks the formation of the starter units (isobutyryl-CoA and 2-methylbutyryl-CoA) of avermectin biosynthesis, with the addition of cyclohexanecarboxylic acid (CHC) to the fermentation broth. While, it can be successfully produced without CHC supplementation if the CHC-CoA biosynthetic gene cassette was introduced into the *bkd* mutant *S. avermitilis* [12]. Furthermore, it has been reported that the replacement of the loading module of avermectin PKS by a cyclohexanecarboxylic unique loading module from phoslactomycin PKS combining with the introduction of the CHC-CoA biosynthetic gene cassette into the wild-type strain *S. avermitilis* led to efficiently produce doramectin without knocking out the *bkd* gene and CHC supplementation [13]. Recently, the biosynthetic gene cluster of milbemycin was characterized by the whole-genome sequencing of *S. bingchenggensis* [14]. The subsequent inactivation of the gene encoding a C5-ketoreductase led to the accumulation of 5-oxomilbemycins A3/A4, which can be used as the substrate for the semi-synthesis of milbemycin oxime through one step chemical reaction [7]. The milbemycin biosynthetic gene cluster *mil* in *S. bingchenggensis* consists of four large ORFs (*milA1*–*milA4*) encoding giant multifunctional polypeptides of PKS, some regulatory genes and genes encoding tailoring enzymes (Additional file 1: Figure S1), which are highly homologous to avermectin biosynthetic gene cluster *ave* [15]. Both milbemycin and avermectin PKSs consist of 12 modules, each of which contains distinctive active site domains catalyzing a specific round of polyketide chain elongation. However, the differences between the module and its counterpart, such as the module 2, the module 7 and the loading module, lead to the structural diversity of avermectin and milbemycin (Additional file 1: Figure S1). Base on the fact that avermectin and milbemycin are similar molecules, it appeared feasible to generate hybrid compounds sharing the structural features of avermectin and milbemycin by combinatorial biosynthesis.

In order to widen the insecticidal spectra of avermectins and milbemycins, it is desirable to generate new derivatives. Herein, ivermectin B1a was produced by the replacement of *ave*DH2-KR2 in the avermectin producer *S. avermilitis* MA-108 with *mil*DH2-ER2-KR2 from *S. bingchenggensis*. The subsequent substitution of *ave*LAT-ACP encoding the loading module of avermectin PKS with *mil*LAT-ACP from *S. bingchenggensis* led to two hybrid compounds 25-methyl and 25-ethyl ivermectin (Fig. 1) simultaneously sharing the structural features of avermectins and milbemycins and showing significantly enhanced insecticidal activity.

## Results

### Construction of aveDH2-KR2 replacement mutant to yield ivermectin

**Fig. 2:**
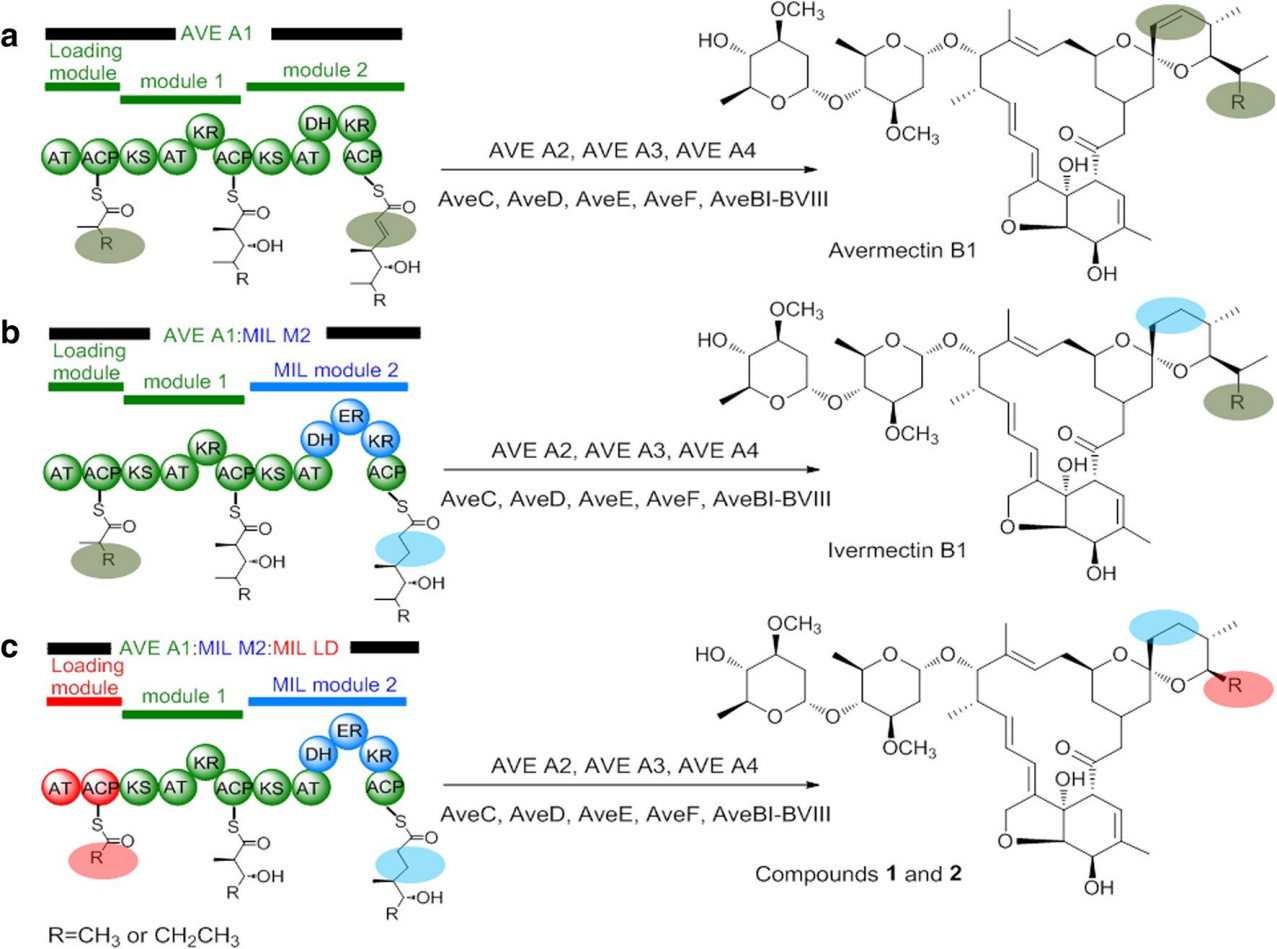
Predicted products of engineered avermectin PKS. **a** AVE A1 of avermectin PKS. **b** AveDH2-KR2 in module 2 of avermectin PKS was replaced with MilDH2-ER2-KR2 of milbemycin PKS. **c** AveDH2-KR2 in module 2 and loading module AveLAT-ACP of avermectin PKS were replaced with MilDH2-ER2-KR2 of and loading module MilLAT-ACP of milbemycin PKS, respectively

**Fig. 3:**
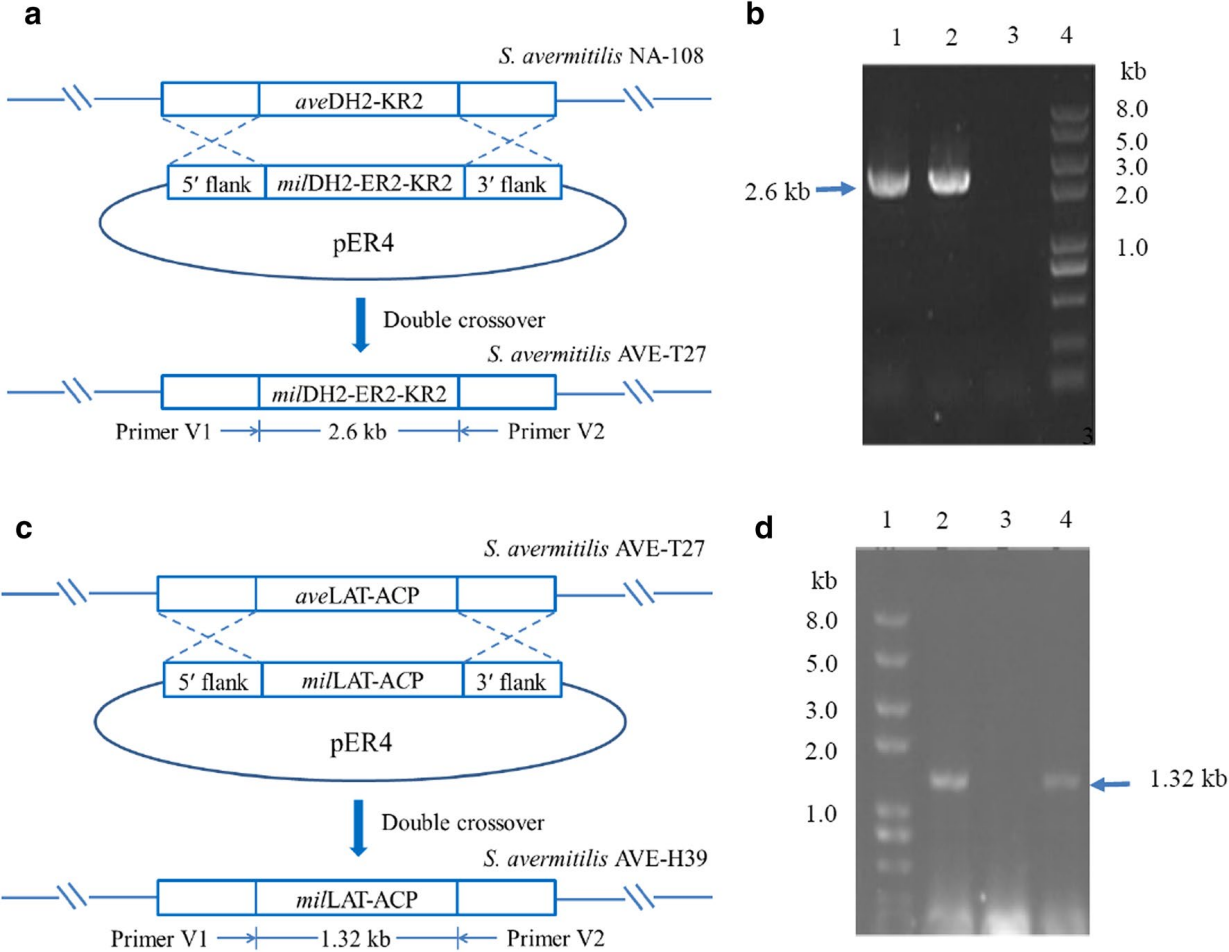
Gene replacement in *S. avermitilis* NA-108. **a** Schematic description of the gene replacement *of ave*DH2-KR2 in *S. avermitilis* NA-108 with *mil*DH2-ER2-KR2 from *S. bingchenggensis* via double crossover. **b** PCR analysis with genomic DNA from *S. avermitilis* NA-108, *S. avermitlis* AVE-T27 and *S. bingchenggensis*, using primers E1 and E2. *Lane 1* *S. bingchenggensis*; *Lane 2* *S. avermitilis* AVE-T27; *Lane 3* *S. avermitilis* NA-108; *Lane 4* DNA ladder. **c** Schematic description of the gene replacement *of ave*LAT-ACP in *S. avermitilis* AVE-T27 with *mil*LAT-ACP from *S. bingchenggensis* via double crossover. **d** PCR analysis with genomic DNA from *S. avermitilis* AVE-T27, *S. avermitlis* AVE-H39 and *S. bingchenggensis*, using primers V1 and V2. *Lane 1* DNA ladder; *Lane 2* *S. bingchenggensis*; *Lane 3* *S. avermitilis* AVE-T27; *Lane 4* *S. avermitilis* AVE-H39

**Fig. 4:**
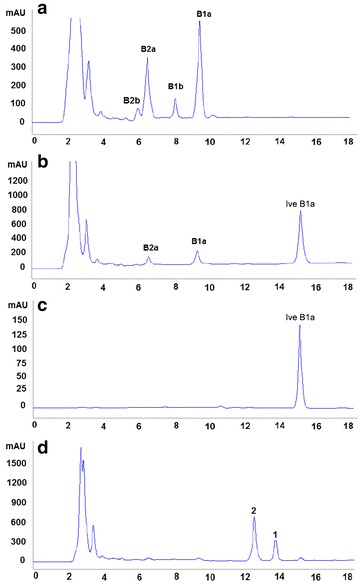
HPLC analysis of the mycelial extracts from the parental and mutant strains. **a** *S. avermitilis* NA-108; **b** *S. avermitlis* AVE-T27; **c** standard sample ivermectin B1a; **d** *S. avermitilis* AVE-H39. *B1a* avermectin B1a, *B2a* avermectin B2a, *B1b* avermectin B1b, *B2b* avermectin B2b, *Ive B1a* ivermectin B1a, *1* compound **1**, *2* compound **2**

On the basis of understanding the biosynthetic pathways of avermectin and milbemycin, we attempted to construct an ivermectin-producing strain by replacing the *ave*DH2-KR2 of avermectin biosynthetic gene cluster in *S. avermitilis* NA-108 with *mil*DH2-ER2-KR2 of milbemycin biosynthetic gene cluster from *S. bingchenggensis* (Fig. 2). PCR verification using the primers E1 and E2 (Additional file 2: Table S1) demonstrated that the expected 2.6-kb DNA fragment encoding MilDH2-ER2-KR2 was obtained from the genomic DNA of *S. bingchenggensis* and the double-crossover mutant *S. avermitilis* AVE-T27, whereas no PCR product was detected from the genomic DNA of *S. avermitilis* NA-108 (Fig. 3a, b). The PCR product was then sequenced, and the results further confirmed that *ave*DH2-KR2 in avermectin biosynthetic gene cluster was successfully replaced with *mil*DH2-ER2-KR2 by double crossover. Compared to the parental strain *S. avermitilis* NA-108, strain AVE-T27 demonstrated a different metabolic profile. As shown in Fig. 4, in addition to the disappearance of avermectin “a” components and the remarkable decrease of avermectin “b” components, a new compound, which showed identical retention time and molecular mass (*m*/*z* = 897, [M+Na]^+^) to those of the authentic sample ivermectin B1a, was detected in culture extracts of *S. avermitilis* AVE-T27 by HPLC analysis. Therefore, the compound was considered to be ivermectin B1a, which was consistent with the designed biosynthetic strategy (Fig. 2).

### Second domain swap to yield new hybrid antibiotics

In order to obtain more avermectin analogs and further investigate the structure–activity relationship of milbemycins and avermectins, the loading module of avermectin PKS encoding by *ave*LAT-ACP in the ivermectin-producing strain *S. avermitilis* AVE-T27 was replaced by that of milbemycin PKS encoding by *mil*LAT-ACP in milbemycin-producing strain *S. bingchenggensis* (Fig. 2). After the successive treatment of the transformants growing at a non-permissive temperature (39 °C) and under non-selective antibiotic pressure, 100 apramycin-sensitive strains were randomly selected and examined by fermentation experiments and HPLC analysis. Compared to strain AVE-T27, 91 apramycin-sensitive strains demonstrated distinct metabolic profiles, in which ivermectin B1a, avermectins B1a and B2a disappeared but two new compounds **1** and **2** with a molecular ion at *m*/*z* = 869.53 [M+Na]^+^ and 855.30 [M+Na]^+^, respectively, were detected (Additional file 3: Figure S2). The double crossover event occurred in strain AVE-H39 with the highest yield of the new compounds was then verified by PCR analysis using a pair of primers V1/V2 (Additional file 2: Table S1), which was designed to amplify the internal fragment of *mil*LAT-ACP. As shown in Fig. 3d, the expected 1.32-kb DNA fragment was obtained from the genomic DNA of *S. bingchenggensis* and the mutant strain AVE-H39, whereas no PCR product was detected from the genomic DNA of AVE-T27. The result implied that the *ave*LAT-ACP in strain AVE-T27 was successfully replaced with *mil*LAT-ACP by double crossover, which was further confirmed by sequencing the PCR product.

### The structure elucidation of hybrid compounds 1 and 2

Compound **1** (Fig. 1) was isolated as a white amorphous powder. Its molecular formula was determined to be C_46_H_70_O_14_ on the basis of HRESIMS at *m*/*z* 869.4652 [M+Na]^+^ (calcd 869.4663 for C_46_H_70_O_14_Na) and ^13^C NMR data (Additional file 4: Table S2). The ^1^H NMR spectrum of compound **1** (Additional file 5: Figure S3) displayed four doublet aliphatic methyls at *δ* 0.82 (d, *J* = 6.3 Hz), 1.15 (d, *J* = 6.9 Hz), 1.25 (d, *J* = 6.1 Hz), 1.27 (d, *J* = 6.4 Hz); two olefinic methyl signals at *δ* 1.50 (s), 1.87 (s); two methoxy signals at *δ* 3.42 (s), 3.43 (s); one *trans*-double bond at 5.72 (overlap), 5.73 (overlap). Its ^13^C NMR (Additional file 6: Figure S4) revealed 46 carbon resonances, including an ester carbonyl carbon at *δ* 174.07, a ketal carbon at 97.63, five *sp*^2^ methines, three *sp*^2^ quaternary carbons, four secondary methyls, two vinylic methyls, one primary methyl, nine methylenes, seventeen aliphatic methines (fourteen oxygenated), one oxygenated quaternary carbon, and two methoxy groups. Comparison of the ^1^H and ^13^C NMR spectral data of **1** with those of avermectin B1b revealed that compound **1** was structurally related to avermectin B1b except for the differences at C22–C23 and C25, where the isopropyl group at C25 and the unsaturated bond between C22 and C23 in avermectin B1b were replaced by an ethyl group and a saturated bond in compound **1**, respectively [16]. The correlations of H-25, H-26 and H-27 in the ^1^H-^1^H COSY spectrum and the HMBC correlations from H-26 to C-25 and C-27 confirmed the presence of an ethyl group at C-25 in compound **1** (Additional file 7: Figure S5). The HMBC correlation between H-22, C-21, C-23 and C-24 indicated the saturated bond between C-22 and C-23. As a consequence, the gross structure of **1** was established as 25-ethyl ivermectin (Fig. 1). Compound **2** was also obtained as a white amorphous powder. Its molecular formula was determined to be C_45_H_68_O_14_ on the basis of HRESIMS at *m*/*z* 855.4508 [M+Na]^+^ (calcd 855.4507 for C_45_H_68_O_14_Na). Comparison of the ^1^H and ^13^C NMR data (Additional file 4: Table S2) with those of **1** indicated that compound **2** and **1** differed only at C25, in that the ethyl group at C-25 in compound **1** was replaced by a methyl group in **2**, which was supported by ^1^H-^1^H COSY correlations of H-25 and H-26 and HMBC correlations from H26 to C24 and C25 (Additional file 7: Figure S5). As a result, the structure of **2** was established as 25-methyl ivermectin (Fig. 1). The 1D- and 2D-NMR spectra of compounds **1** and **2** can be found in the supplemental material (Additional files 5, 6, 7, 8, 9, 10, 11, 12, 13, 14, 15: Figures S3–S13).

### The bioactivity of hybrid compounds 1 and 2

The insecticidal activity of compounds **1** and **2** against *C. elegans* and *M. separate* was evaluated and compared to that of commercial insecticides milbemycin and ivermectin. The results demonstrated that the lethal effects of the tested compounds were enhanced with increased concentrations, indicating that the insecticidal activity was dose-dependent. However, the mixture of **1** and **2** possessed higher insecticidal activities against *C. elegans* and *M. separate* than milbemycin and ivermectin. As listed in Table 1, the LC_50_ of the mixture of **1** and **2** is 2.2 ± 0.7 μg/ml, which is approximately 2.5-fold and 4.6-fold lower than that of milbemycin (5.42 ± 2.1 μg/ml) and ivermectin (10.1 ± 1.3 μg/ml), respectively. Moreover, it exhibited impressive insecticidal activity against *M. separata* with a LC_50_ of 19.4 ± 1.3 μg/ml, which is 5.7-fold lower than that of milbemycin (110.7 ± 7.4 μg/ml).

**Table 1:** Insecticidal activity of the mixture of 1 and 2 compared with those of milbemycins A3/A4 and ivermectin

Samples	LC_50_ (μg/ml)^c^	
	*C. elegans*	*M. separata*
The mixture of **1** and **2** ^a^	2.2 ± 0.7	19.4 ± 1.3
Milbemycin A3/A4^b^	5.4 ± 2.1	110.7 ± 7.4
Ivermectin	10.1 ± 1.3	

^a^The mixture contains 70 % compound **1** and 30 % compound **2**

^b^Milbemycins A3/A4 contains 70 % A4 and 30 % A3

^c^Values are the means ± SDs of three independent experiments

### Genetic stability of AVE-T27 and AVE-H39

The genetic stabilities of strains AVE-T27 and AVE-H39 to produce ivermectin B1a and compounds **1** and **2** were evaluated by five successive subcultivation tests. The shaking flask experiments and HPLC analysis showed that the yield of ivermectin B1a and 25-methyl/25-ethyl ivermectin produced by AVE-T27 and AVE-H39 among five generations ranged from 3450 ± 65 to 3510 ± 37 μg/ml and 3045 ± 78 to 3087 ± 46 μg/ml, respectively. These results suggested that strains AVE-T27 and AVE-H39 were genetically stable and could be applied in industry.

## Discussion

Since the discovery of avermectin in 1975, many efforts have been devoted to its structural modification to yield avermectin derivatives. To date, six avermectins (abamectin, ivermectin, doramectin, emamectin, eprinomectin and selamectin) have been commercialized and widely used as anthelmintic and insecticidal drugs in animal health and agriculture [17]. The structural differences of these drugs are located on C25, C22–C23 and C13 positions. For example, ivermectin (22,23-dihydroavermectin B1), which is synthesized by chemical reduction of the double bond between C22 and C23 of avermectins B1, exhibits the same effective antiparasitic activity but lesser toxic side effect than avermectins B1 [18]. Doramectin (C25-cyclohexyl avermectin B1), an avermectin analog with a cyclohexyl group at C25, demonstrates better pharmacokinetic properties and efficacy than avermectin [19]. Compared to avermectin, eprinomectin, emamectin and selamectin possess different groups at C13 and show unique pharmacokinetic characteristics. The various applications of avermectins in agricultural and veterinary fields have attracted much attention, and more avermectin analogs with high insecticidal and acaricidal activity have been reported [20, 21]. Milbemycins are a group of 16-membered macrolides chemically related to avermectins with the major structural differences at C25, C22–C23 and C13. Due to the high and broad-spectrum activity against insects and parasites, low toxicity to mammals, and environment friendliness, milbemycins have received considerable interest in agricultural chemistry, and four kinds of milbemycins including milbemectin (a mixture of milbemycins A3/A4), milbemycin oxime, lepimectin, and latidectin have been marketed and used as a broad-spectrum acaricide, anthelmintic and insecticide [7, 22]. Although the natural avermectins and milbemycins demonstrate structural differences, some commercial avermectins and milbemycins share the same structural features. For example, selamectin and ivermectin contain a saturated bond at C22–C23, which is similar to milbemycins; while latidectin and lepimectin contain 13-substituted groups, the modified pattern of which is different from milbemectin and milbemycin oxime, but similar to avermectins. Inspired by the structure–activity relationship of avermectins and milbemycins, series of compounds with the partial structural features of milbemycins and avermectins were synthesized and showed potential insecticidal activity [23].

The polyketide backbond of avermectin and milbemycin is biosynthesized by the multifunctional and multimodular proteins referred as type I polyketide synthetases (type I PKSs). The type I PKSs consist of several modules, each of which always carries three core domains acyltransferase (AT), acyl carrier protein (ACP) and ketosynthase (KS) as well as other accessory domains KR, DH and ER [24]. The organization of the modular PKSs allows a wide range of complex polyketide products to be assembled from simple precursors. Given the increased understanding of biosynthetic machineries of polyketides, combinatorial biosynthesis by rearranging the domains in PKSs has been developed as an alternative strategy to generate novel unnatural polyketides [25, 26]. Although milbemycin biosynthetic gene cluster *mil* and avermectin biosynthetic gene cluster *ave* demonstrate different gene organizations, both of them contain four large ORFs (*milA1*–*milA4*/*aveA1*–*aveA4*), a regulatory gene (*milR*/*aveR*) and some genes encoding tailoring enzymes (*milC*/*aveC*, *milE*/*aveE*, *milF*/*aveF*, *milD*/*aveD*) (Additional file 1: Figure S1). The four large ORFs encode the PKS, which is responsible for the biosynthesis of polyketide backbond and consists of a loading module and 12 elongating modules. These homologous ORFs in *mil* and *ave* share high identity in protein sequences ranging from 48.41 to 64.78 %, leading to a similar polyketide backbone observed in milbemycin and avermectin. However, the differences at the loading module, module 2 and module 7 are believed to be associated with the structural changes at C25, C22–C23 and C13 positions. Inspired by the structural diversity and impressive bioactivity of milbemycins and avermectins, two new avermectin derivatives were designed by reconstructing the biosynthetic pathway of avermectins (Fig. 2). Firstly, the *ave*DH2-KR2 of avermectin biosynthetic gene cluster in *S. avermitilis* NA-108 was replaced by *mil*DH2-ER2-KR2 of milbemycins biosynthetic gene cluster from *S. bingchenggensis* to yield the ivermectin-producing strain AVE-T27. Compared to the parental strain NA-108, strain AVE-T27 no longer produced “b” components of avermectins (Fig. 4b), and the yield of avermectin B1a and B2a (259 ± 23 and 173 ± 18 μg/ml, respectively)decreased approximately 7 fold and 2.3 fold, respectively. The remarkable decrease in the yield of avermectins was also observed in other ivermectin-producing strains [6, 8, 11]. It is obvious that strain NA-108 only produces four components of avermectins, of which the “a” components B1a and B2a are produced in quantity (Fig. 4a), suggesting that the loading module of the avermectin PKS in strain NA-108 may dominantly utilize 2-methylbutyryl as the starter unit for polyketide assembly. Therefore, the substitution of *ave*DH2-KR2 by *mil*DH2-ER2-KR2 resulted in the substantial accumulation of ivermectin B1a, whereas the yield of ivermectin B1b may be too low to be detected (Fig. 4b). Before the thorough understanding the function of AveC in avermectin biosynthesis [27], the unsaturated bond of C22–C23 was considered to be formed through the optional dehydration by partially active dehydratase (DH) in module 2 with the assistance of AveC [28]. Therefore, several attempts have been made to construct ivermectin-producing strains by replacing the partially active DH with completely active DH and ER from other PKSs through the genetic manipulation of avermectin biosynthetic gene cluster. However, the yield of ivermectin produced by the genetic engineered strains is too low for large-scale production probably due to the poor folding of the hybrid PKSs and unfavorable protein–protein interactions [6, 8, 11]. Thus, ivermectin is still synthesized by a regiospecific hydrogenation at C22–C23 of avermectins using rhodium chloride as the catalyst, even though this process suffers from expensive cost and heavy metal pollution [6]. It is worth noting that strain AVE-T27 demonstrated excellent performance in producing ivermectin B1a with a yield of 3450 ± 65 μg/ml, which is only slightly lower than the total amount of avermectins (3577 ± 26 μg/ml) produced by the parental strain NA-108. It is commonly reported that PKS bioengineering experiments often produce new compounds at compromised yields [6, 8, 10, 11, 24, 29]. For examples, the total amount of avermectins and ivermectins produced in the ivermectin-producing strains Ive12-4 and OI-31, which were constructed by replacing the AveDH2-KR2 in *S. avermitilis* Olm73-12 with PikDH4-ER4-KR4 from pikromycin PKS and OlmDH3-ER3-KR3 from oligomycin PKS, respectively, was estimated to be only 1–3 % of the amount of avermectins produced by the parental strain or decreased approximately eightfold [6, 8]. Compared to PikDH4-ER4-KR4 (46.48 %) and OlmDH3-ER3-KR3 (43.06 %), MilDH2-ER2-KR2 demonstrates a higher identity (51.50 %) with AveDH2-KR2. Furthermore, both of MilDH2-ER2-KR2 and AveDH2-KR2 catalyze the reduction of C22–C23 in the similar polyketide backbone. Therefore, it is no surprise that the substitution of AveDH2-KR2 with MilDH2-ER2-KR2 could not lead to the drastic decrease in the total amount of avermectins and ivermectin. Indeed, the use of alternative domains and modules from PKSs of structurally related polyketides is considered as an efficient approach to generate hybrid PKSs with high yield [30]. The high yield of ivermectin B1a produced by strain AVE-T27 implied that the DH2-ER2-KR2 from milbemycin PKS may be more suitable for swapping AveDH2-KR2 than the domains from other PKS reported previously [6, 8, 11]. We speculated that the domain swapping between AveDH2-KR2 and MilDH2-ER2-KR2 does not seriously affect the substrate specificity of the KS domain and dynamic KS:ACP interaction, which are believed to play crucial roles in polyketide chain transfer and elongation [10, 24]. However, strain AVE-T27 still produced a small amount of avermectins B1a and B2a, suggesting the incompletable function of MilDH2-ER2-KR2 in hybrid PKS. Recently, Sun et al. have revealed a dual function of AveC, which works as a spirocyclase and an independent dehydratase, involved in the spiroketal formation and the optional dehydration of C22–C23 during the avermectin biosynthesis [27]. Due to the complete inactivity of AveDH2, AveC was believed to be the only factor responsible for C22-C23 dehydration to yield the “1” components of avermectins. This dehydration reaction is post-PKS but precedes spiroketal formation, and the spirocyclase activity of AveC can tolerate variation of the C22-C23 bond. Therefore, we assumed that parts of avermectin B1a produced by AVE-T27 are formed by the incompletable activity of MilDH2-ER2-KR2 during skeleton assembly process, while the others are probably associated with the optional dehydratase activity of AveC in the post-PKS modification.

Loading modules of type I PKSs catalyze selection and recruitment of starter units into polyketides. Accordingly, starter units are incorporated into polyketides at only one position, and swapping of the loading module can lead to changes in the side chain originating from the starter unit [24, 31]. For example, the replacement of the loading module of the erythromycin PKS or spinosyn PKS by that of avermectin PKS resulted in the production of novel erythromycin or spinosyn analogs [32, 33]. Recently, it has been reported that the replacement of the loading module of avermectin PKS with that of phoslactomycin PKS, which prefers cyclohexylcarbonyl-CoA as the start unit, led to enhanced ratio of doramectin to avermectins produced by genetic engineered *S. avermitilis* [13]. Therefore, the loading module of avermectin PKS could be a suitable site for module swapping to generate unnatural avermectins. After the replacement of the loading module of avermectin PKS in strain AVE-T27 by that of milbemycin PKS, two new avermectin derivatives with a methyl or ethyl group at C25 position were obtained (Fig. 1). The loading module of avermectin PKS possesses broad substrate specificity, for example, it can use 2-methylbutyryl-CoA, isobutyryl-CoA and cyclohexylcarbonyl-CoA as substrates to biosynthesize avermectin “a” components, avermectin “b” components and doramectin, respectively [2, 34]. However, the loading module of milbemycin in *S. bingchenggensis* seems to only use acetyl-CoA and propionyl-CoA as substrates to biosynthesize 25-methyl and 25-ethyl milbemycins, respectively [15]. Therefore, it was speculated that the loading module of avermectin PKS possesses a broader substrate specificity than that of milbemycin PKS. Compared to the yield of ivermectin produced by AVE-T27, the total amount of 25-methyl and 25-ethyl ivermectin (3045 ± 78 μg/ml) produced by AVE-H39 is satisfory. However, no avermectins with C25-isopropyl, C25-isobutyl groups were detected (Fig. 4d), which confirmed the relatively narrow substrate specificity of the loading module of milbemycin PKS. Although the loading module of avermectin PKS exhibits an identity of 46.54 % with that of milbemycin PKS on protein level, it seems that the domain swapping between them did not seroiusly affect the biosynthetic ability of the resultant hybrid PKS. The high yield of 25-methyl and 25-ethyl ivermectin again confirmed that the use of alternative modules from PKSs of structurally related polyketides is an efficient approach to generate hybrid polyketides.

The previous structure–activity relationship studies of avermectins suggested that C5, C13 and C22–C23 are essential for the bioactivity, and the subsequent modification at these positions successfully developed several commercial drugs such as ivermectin, emamectin, eprinomectin and selamectin [17]. Although some C25-substitituted avermectins were designed [34–36], only doramectin with a cyclohexyl group at C25 was commercialized. Compared to ivermectin, compounds **1** and **2** exhibited significantly enhanced insecticidal activity, implying that the C25 is a potential target for structural modification, which provides new clues for the structural modification of avermectins.

## Conclusions

In conclusion, 25-methyl and 25-ethyl ivermectin were obtained by domain swapping. The impressive insecticidal activity of these two new compounds suggested their promising use as novel insecticides in agriculture. Furthermore, the high yield and genetic stability of the engineered strains *S. avermitilis* AVE-T27 and AVE-H39 highlight their potential in industrial production of ivermectin B1a and 25-methyl/25-ethyl ivermectin, respectively.

## Materials and methods

### Strains, vectors, reagents, and cultivation

All bacterial strains and plasmids used in this study are listed in Table 2. The initial strain *S. avermitilis* NA-108, originating from the mutation of *S. avermitilis* NEAU1069, can only produces “B” components of avermectins with high yield but does not produce “A” components of avermectins and oligomycin. *S. avermitilis* NEAU1069 and *S. bingchenggensis* have been deposited at the China General Microbiology Culture Collection Center, Institute of Microbiology, Chinese Academy of Science with accession numbers of CGMCC2943 and CGMCC1734, respectively. The 16S rDNA sequence of *S. avermitilis* NEAU1069 and *S. bingchenggensis* was deposited in GenBank with accession numbers of DQ768097 and DQ449953, respectively. Primers (Additional file 2: Table S1) were synthesized in Sangon Biotech (Shanghai, China). Restriction endonucleases, DNA ligase, and DNA polymerases were purchased from TaKaRa Biotechnology (Dalian, China). DNA sequencing was performed by GenScript (Nanjing, China). MS (mannitol soya flour) medium was used for *S. avermilitis* NA-108 sporulation and conjugation between *Escherichia coli* and *Streptomyces*. YEME (yeast extract-malt extract) medium containing 25 % sucrose was used to grow mycelia for the isolation of total DNA [37]. Luria–Bertani (LB) medium was used for *E. coli* propagation [38]. All *E. coli* procedures were performed according to standard protocols [38]. Isolation of genomic DNA from *S. avermitilis* NA-108 and agarose gel electrophoresis were performed according to the standard methods [37, 38].

**Table 2:** Strains and plasmids used in this study

Strains/plasmids	Relevant characteristics^a^	References
Strains		
*E. coli*		
DH5α	Host strain for cloning	Invitrogen
ET12567/pUZ8002	Donor strain for conjugation	Kieser et al. [37]
*S. bingchenggensis*	The milbemycin-producing strain	Wang et al. [14]
*S. avermitilis*		
NA-108	Mutant strain of *S. avermitilis* NEAU1069 producing “B” components of avermectins as main secondary metabolites	This study
AVE-T27	Mutant strain of NA-108 with the replacement of *ave*DH2-KR2 by *mil*DH2-ER2-KR2	This study
AVE-H39	Mutant strain of AVE-T27 with the replacement of *ave*LAT-ACP by *mil*LAT-ACP	This study
Plasmids		
pUC19	Cloning vector for *E. coli*, *Amp* ^*R*^	TaKaRa
pKC1139	*E. coli*-*Streptomyces* shuttle vector, *Am* ^*R*^	Kieser et al. [37]
pER-1	pUC19 derivative containing *ave*DH2-KR2 upstream fragment, *Amp* ^*R*^	This study
pER-2	pUC19 derivative containing *ave*DH2-KR2 upstream and downstream fragments, *Amp* ^*R*^	This study
pER-3	pUC19 derivative containing *mil*DH2-ER2-KR2 together with *ave*DH2-KR2 upstream and downstream fragments, *Amp* ^*R*^	This study
pER-4	pKC1139 derivative containing *mil*DH2-ER2-KR2 together with *ave*DH2-KR2 upstream and downstream fragments, *Am* ^*R*^	This study
pLM-1	pMD19-T derivative containing *mil*LAT-ACP together with *ave*LAT-ACP upstream and downstream fragments, *Amp* ^*R*^	This study
pLM-2	pKC1139 derivative containing *mil*LAT-ACP together with *ave*LAT-ACP upstream and downstream fragments, *Amp* ^*R*^	This study

^a^ *Amp* ^*R*^ ampicillin resistance, *Am* ^*R*^ apramycin resistance

### Construction of *ave*DH2-KR2 replacement mutant strain

In order to replace the DNA region encoding DH2-KR2 of avermectin PKS (*ave*DH2-KR2) with the DNA fragment encoding DH2-ER2-KR2 of milbemycin PKS (*mil*DH2-ER2-KR2), recombinant plasmid pER-4 was constructed. Using genomic DNAs of *S. avermitilis* NA-108 as template, a 1106-bp fragment upstream of the *ave*DH2-KR2 was amplified with primers a1 and a2. The amplified fragment was digested with HindIII/XbaI and ligated into corresponding sites of pUC19 to generate pER-1. Then, a 1055-bp fragment downstream of the *ave*DH2-KR2 amplified with primers c1 and c2 was digested with XbaI/EcoRI and then ligated to the corresponding sites of pER-1 to yield pER-2. The introduction of XbaI site did not change the natural amino acids sequence at the linker regions. The *mil*DH2-ER2-KR2 (3120-bp) fragment was amplified with primers b1 and b2 using genomic DNAs of *S. bingchenggensis* as template, and the resultant PCR products were cloned into the XbaI sites of pER-2 to give pER-3. The 5.27-kb insert was recovered from pER-3 by digesting with HindIII/EcoRI and inserted into the same sites of pKC1139 to generate pER-4. After the verification by PCR amplification and restriction digestion analysis, pER-4 was transformed into the non-methylating *E. coli* ET12567/pUZ8002. The conjugations were performed using the spores of *S. avermitilis* NA-108 according to the literature [16] and exconjugants were selected using MS agar containing apramycin. These cultures were grown at 28 °C for 2 days, then at 39 °C for 7–10 days. The colonies that were apramycin-resistant at 39 °C were identified as the integrating mutants, in which a single-crossover homologous recombination event took place. Insertion mutants were confirmed by PCR analysis and inoculated on nonselective MS plates at 28 °C for a second round of recombination. Double crossover mutants were screened by replica from the colonies grown on the MS medium. Mutants that lost resistance to apramycin were selected for further screening and genotypic confirmation by PCR using E1 and E2 as primers. The obtained double-crossover mutant was designated as AVE-T27.

### Construction of *ave*LAT-ACP replacement mutant strain

To construct plasmid pLM-2 for replacement of the gene encoding the loading module of avermectin PKS (*ave*LAT-ACP) with that of milbemycin PKS (*mil*LAT-ACP), the overlap extension PCR was employed [39]. Firstly, three pairs of primers A1/A2, B1/B2 and C1/C2 were designed to amplified 5′flank of *ave*LAT-ACP (1008-bp), *mil*LAT-ACP (1407-bp) and 3′flank of *ave*LAT-ACP (1022-bp), respectively. Then a two-step PCR was employed: in the first round PCR, three fragments were mixed in a ratio of 1:3:1 (total DNA amounts about 250 ng), with 0.2 mM of each dNTP, 0.5 U PrimeSTAR HS DNA polymerase (Takara, Dalian, China), 1× PrimeSTAR Buffer (Mg^2+^ plus), and 10 % DMSO, the PCR program consisted of 12 repetitive cycles with a denaturation step at 94 °C for 30 s, an annealing step at 57 °C for 15 s and an elongation step at 72 °C for 3 min; in the second round PCR, 4 μl reactants from above was took as template, primers A1/C2 and other reagents were added, the PCR program consisted of 25 repetitive cycles with a denaturation step at 94 °C for 30 s, an annealing step at 60.6 °C for 15 s and an elongation step at 72 °C for 4 min. A 3400-bp fragment was obtained and ligated to pMD19-T (Takara, Dalian, China) to generate pLM-1. After sequencing and PCR analysis, the 3.4-kb fusion fragment was recovered from pLM-1 by digesting with HindIII/XbaI and inserted into the same sites of pKC1139 to generate pLM-2. Following the procedure described above, pLM-2 was introduced into strain AVE-T27 for a second replacement. After the validation by PCR amplification using V1 and V2 as primers, the obtained double-crossover mutant was designated as AVE-H39.

### Fermentation and HPLC analysis of antibiotic production

*S. avermitilis* NA-108 and all genetically engineered strains were fermented under the same culture condition. The strains were firstly cultured in seed medium (corn starch 2 %, glucose 0.5 %, yeast extract 1 %, cottonseed cake 1 %, and CoCl_2_·6H_2_O 0.005 %, pH 7.2) at 28 °C for 42 h on a rotary shaker at 250 rpm. Then 2.0 ml of the culture was transferred into 250-ml Erlenmeyer flasks containing 25 ml of the fermentation medium consisting of corn starch 80 g/l, glucose 5 g/l, peptone10 g/l, cottonseed cake 10 g/l, NaCl 1 g/l, K_2_HPO_4_·3H_2_O 1 g/l, MgSO_4_·7H_2_O 1 g/l, CaCO_3_ 1 g/l and CoCl_2_·6H_2_O 5 mg/l, pH 7.0. Fermentation was carried out at 28 °C for 8 days on a rotary shaker at 250 rpm. After finishing the fermentation, the broths were mixed with an equal volume of methanol. The resultant mixture was then centrifuged at 12,000×*g* for 20 min, and the supernatant was filtered through a 0.22 μm membrane filter and analyzed by HPLC. HPLC was performed with a Shimadzu LC-2010CHT system (Shimadzu, Koyoto, Japan) by using a NOVA-PAK^R^ C18 column (3.9 × 150 mm, 5 μm, Waters, Milford, MA) at a flow rate of 1.0 ml/min, CH_3_OH/H_2_O (85:15, v/v) and detected at 246 nm. For LC/MS analysis, the positive electrospray ionization mass spectra were obtained from a Waters Q-TOF Micro LC–MS-MS spectrometer (electrospray voltage, 3.5 kV; heated capillary temperature, 450 °C; gas flow, 900 l/h) coupled with a Agilent HPLC 1200 system equipped with a Eclipse × DB-C18 column (4.6 × 150 mm, 5 μm) using a mobile phase of CH_3_OH/H_2_O (80:20, v/v). The flow rate was 0.3 ml/min, and the detection was at 246 nm.

### Purification of hybrid antibiotics

The hybrid antibiotics produced by the recombinant strain AVE-H39 were purified from the culture broth. After the fermentation, the broth (5 l) was centrifugated, and the resulting cake was washed with H_2_O. MeOH (10 l) was used to extract the washed cake. The MeOH extract was evaporated under reduced pressure to approximately 0.2 l at 45 °C and the resulting concentrate was extracted three times using an equal volume of EtOAc. The combined EtOAc phase was concentrated under reduced pressure to yield oily substances. The residual oily substance was chromatographed on silica gel and eluted with a petroleum ether-acetone mixture (95:5–50:50, v/v). The fractions eluted with the petroleum ether-acetone mixture (95:5–75:25, v/v) were combined and evaporated to obtain a crude mixture. The crude mixture was then subjected to semipreparative HPLC (Agilent 1100, Zorbax SB-C18, 5 μm, 9.4 × 250 mm; 1.5 ml/min; 246 nm) by eluting with CH_3_OH-H_2_O (80:20, v/v) to afford compound **1** and **2**.

### Structural analysis and bioactivity of hybrid antibiotics

The HRESI-MS spectra were taken on a Q-TOF Micro LC–MS-MS spectrometer (Waters, Milford, MA, USA) and an API QSTAR time-of-flight spectrometer, respectively. 1D- and 2D-NMR spectra were recorded on Bruker AM-400 and DRX-600 spectrometers (Bruker, Rheinstetten, Germany) using 2.5 mm microcells (Synthware) for CDCl_3_. The 7.26 ppm and 77.23 ppm resonance of CDCl_3_ was used as internal references for ^1^H NMR spectra and ^13^C NMR spectra, respectively. The insecticidal activity of the mixture of compounds **1** and **2** against the second-instar larva of *M. separata* was measured by the leaf-dipping method [17]. Briefly, the test sample was dissolved in methanol at a concentration of 100 μg/ml and diluted to appropriate concentrations with distilled water containing 1 % Tween 80. Wafer discs (1-cm diamemter, 1-mm-thick) made from fresh wild corn leaves were dipped into the diluted solutions of the test sample for 3 s, and then taken out and kept in a conditioned room. The number of dead larvae was recorded at 48 h after treatment, and the corrected mortality was calculated. The insecticidal activity of the test sample against *C. elegans* was evaluated according to methods described previously [40]. The appropriate amounts of the diluted solutions of the test sample were added to 1-l portion of an aqueous suspension containing living nematodes *C. elegans*. The mixtures were kept at 25 °C for 15 h after shaking. The number of nematodes that were immobilized and the total number of nematodes tested were counted under a stereoscopic microscope. Immobilized rates against the total number of tested nematodes were calculated. The insecticidal activity was expressed as LC_50_ values, which were calculated by the LOGIT method. Milbemycin A3/A4 (A3:A4 = 3:7) and ivermectin were used as positive controls.

## Additional files

**Additional file 1:** Figure S1. Organizations of avermectin and milbemycin biosynthetic gene clusters.
**Additional file 2:** Table S1. Primers used for gene cloning, constructing and confirming the mutants.
**Additional file 3:** Figure S2. The ESI-MS spectra of compounds (a) **1** and (b) **2**.
**Additional file 4:** Table S2. ^1^H and ^13^C NMR data of compounds **1** and **2**.
**Additional file 5:** Figure S3. ^1^H NMR spectrum of compound **1** in CDCl_3_ (400 MHz).
**Additional file 6:** Figure S4. ^13^C NMR spectrum of compound **1** in CDCl_3_ (100 MHz).
**Additional file 7:** Figure S5. The ^1^H-^1^H COSY and HMBC correlations of compounds **1** and **2**.
**Additional file 8:** Figure S6. COSY spectrum of compound **1**.
**Additional file 9:** Figure S7. HSQC spectrum of compound **1**.
**Additional file 10:** Figure S8. HMBC spectrum of compound **1**.
**Additional file 11:** Figure S9. ^1^H NMR spectrum of compound **2** in CDCl_3_ (400 MHz).
**Additional file 12:** Figure S10. ^13^C NMR spectrum of compound **2** in CDCl_3_ (100 MHz).
**Additional file 13:** Figure S11. COSY spectrum of compound **2**.
**Additional file 14:** Figure S12. HSQC spectrum of compound **2**.
**Additional file 15:** Figure S13. HMBC spectrum of compound **2**.
