# Supplementary figures and images for: Designed biosynthesis of 25-methyl and 25-ethyl ivermectin with enhanced insecticidal activity by domain swap of avermectin polyketide synthase
Source: Microb Cell Fact. 2015 Sep 24;14:152. doi: 10.1186/s12934-015-0337-y (PMC4581413; doi:10.1186/s12934-015-0337-y)

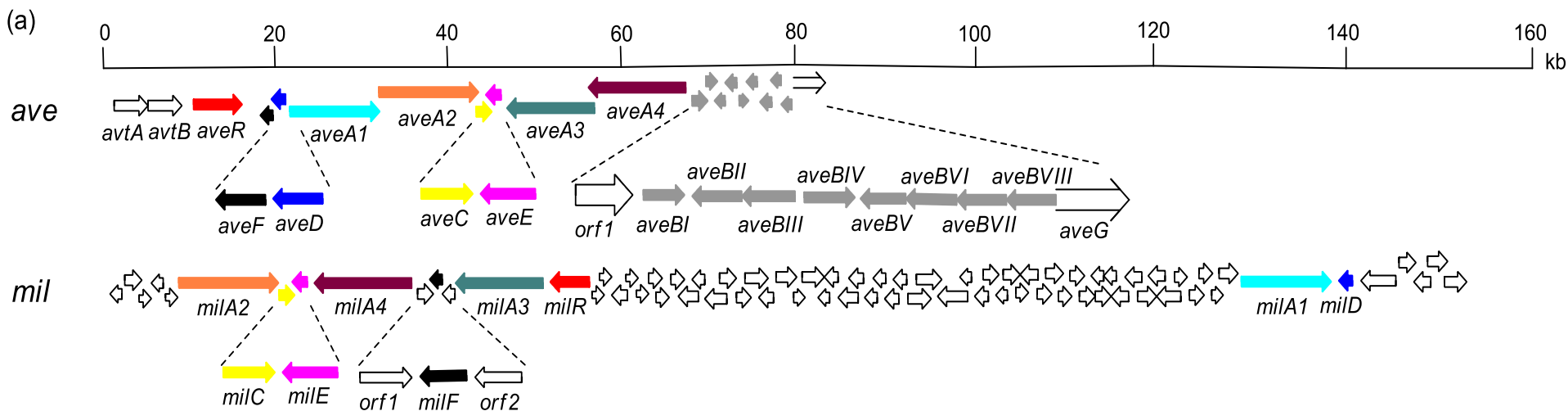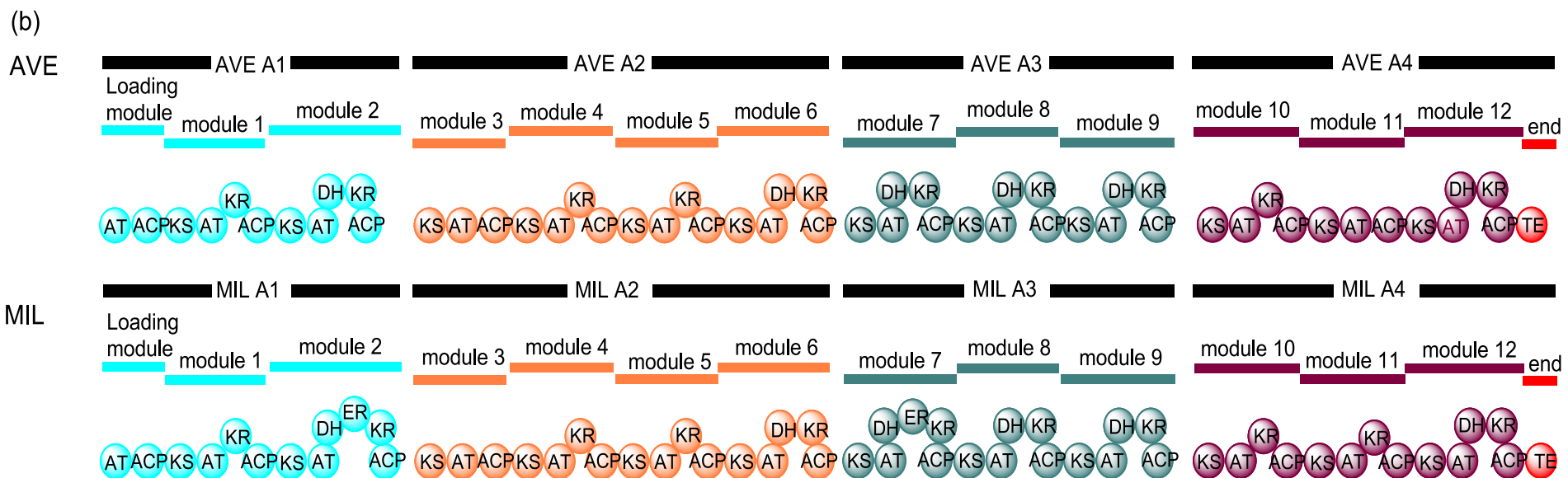

Supplement: Supplementary file 1 — Additional file 1: Figure S1. Organizations of avermectin and milbemycin biosynthetic gene clusters. [file 12934_2015_337_MOESM1_ESM.pdf]

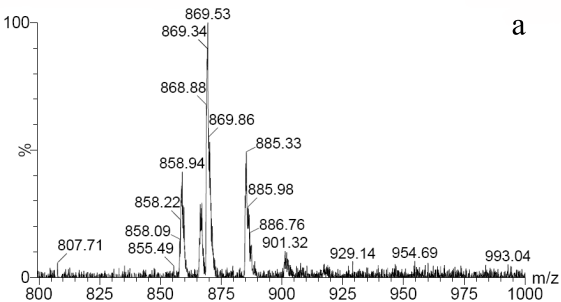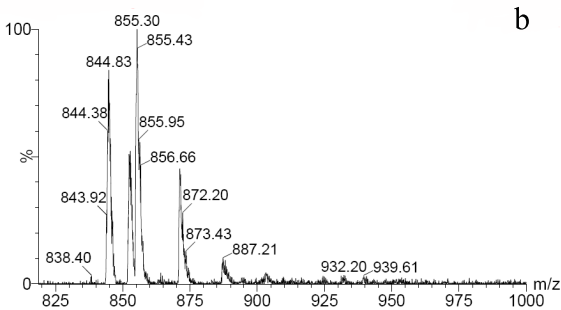

Supplement: Supplementary file 3 — Additional file 3: Figure S2. The ESI-MS spectra of compounds (a) 1 and (b) 2. [file 12934_2015_337_MOESM3_ESM.pdf]

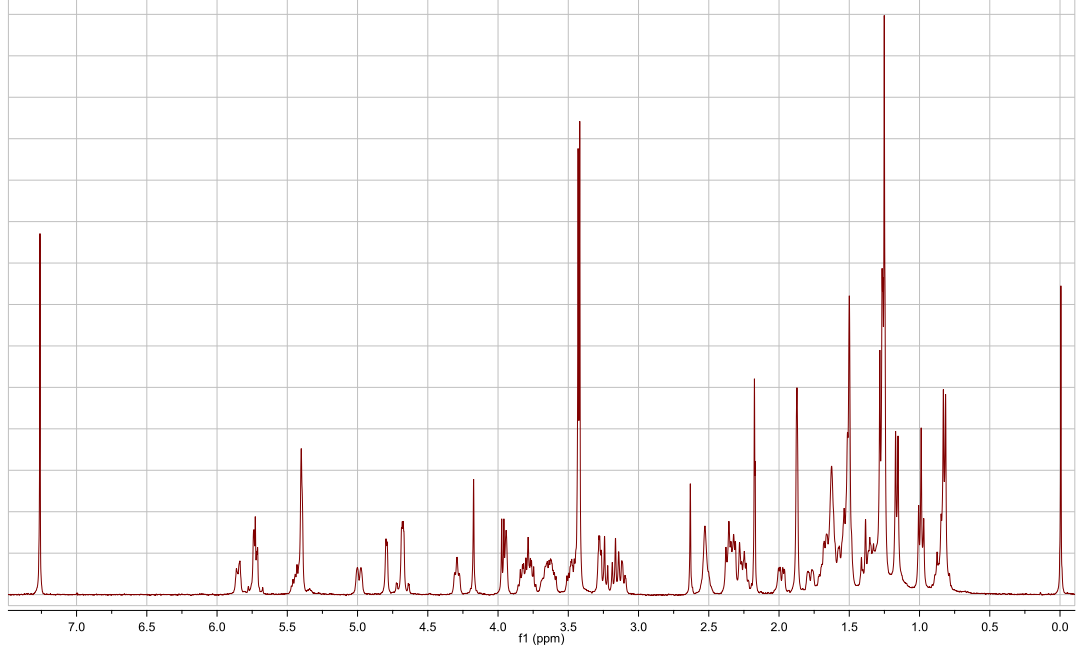

Supplement: Supplementary file 5 — Additional file 5: Figure S3. 1H NMR spectrum of compound 1 in CDCl3 (400 MHz). [file 12934_2015_337_MOESM5_ESM.docx]

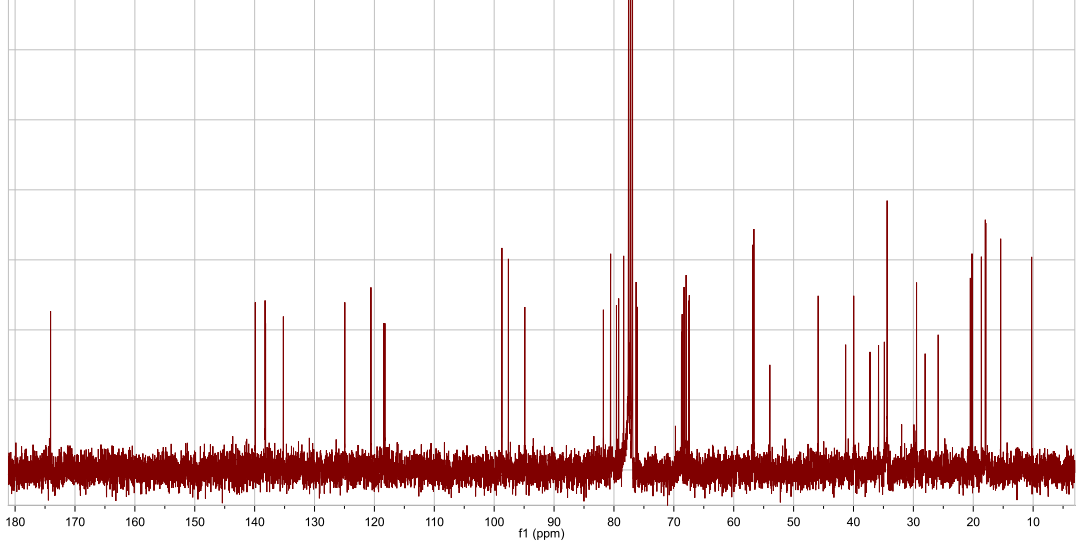

Supplement: Supplementary file 6 — Additional file 6: Figure S4. 13C NMR spectrum of compound 1 in CDCl3 (100 MHz). [file 12934_2015_337_MOESM6_ESM.docx]

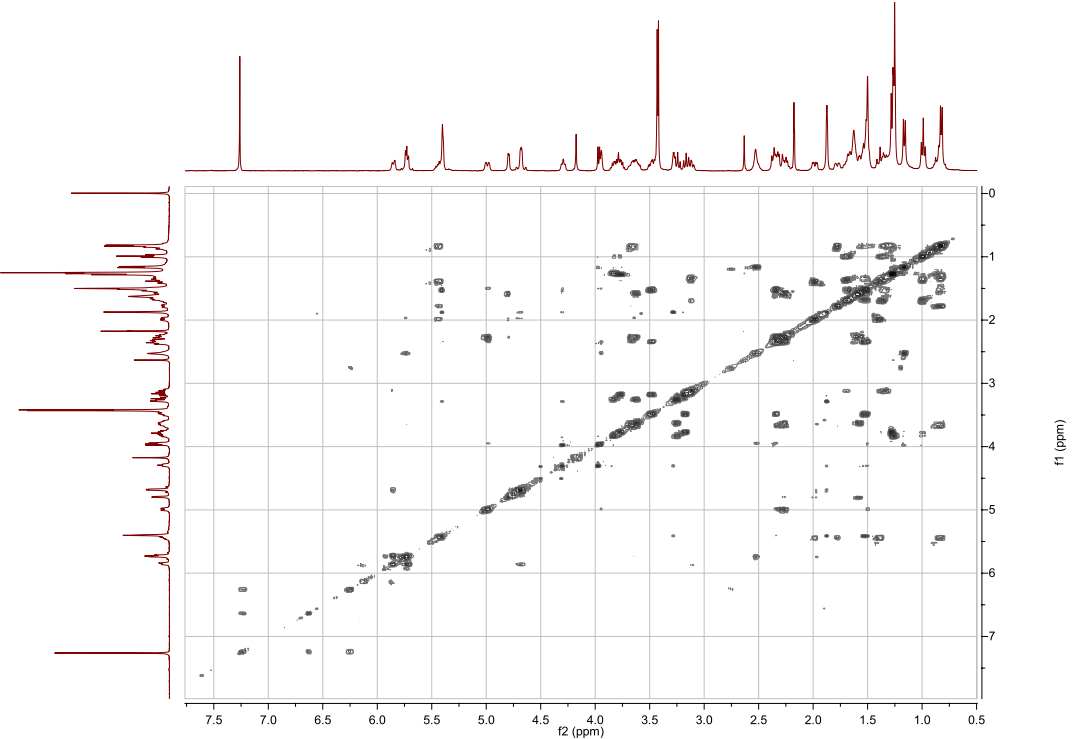

Supplement: Supplementary file 8 — Additional file 8: Figure S6. COSY spectrum of compound 1. [file 12934_2015_337_MOESM8_ESM.docx]

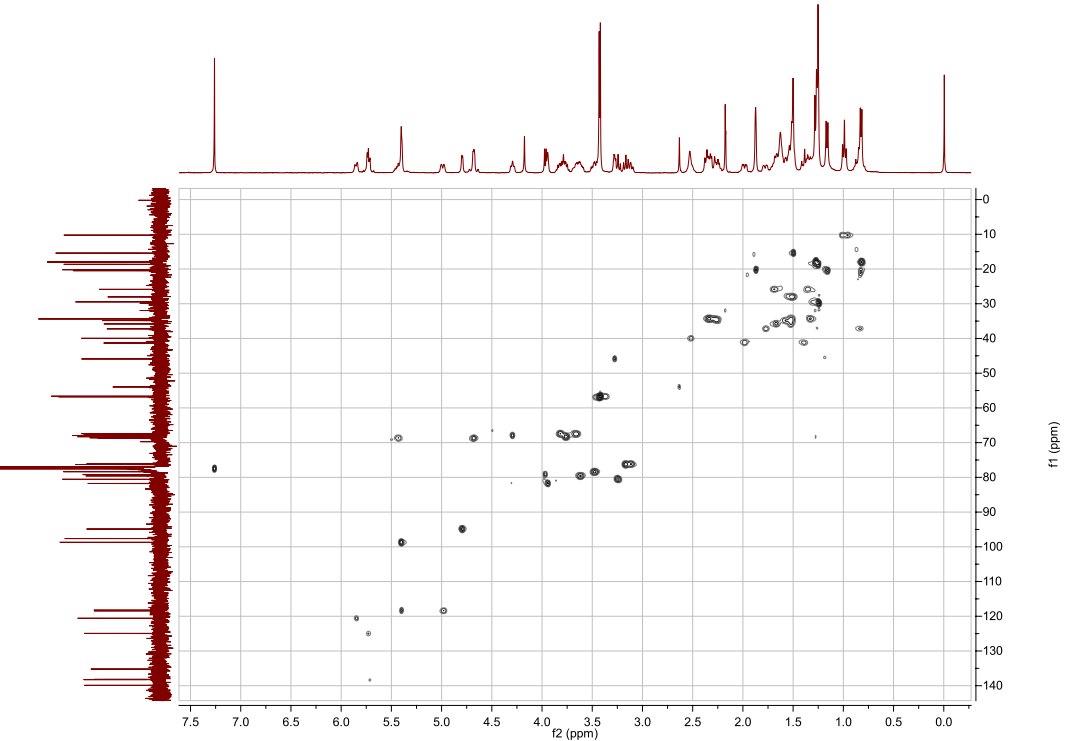

Supplement: Supplementary file 9 — Additional file 9: Figure S7. HSQC spectrum of compound 1. [file 12934_2015_337_MOESM9_ESM.docx]

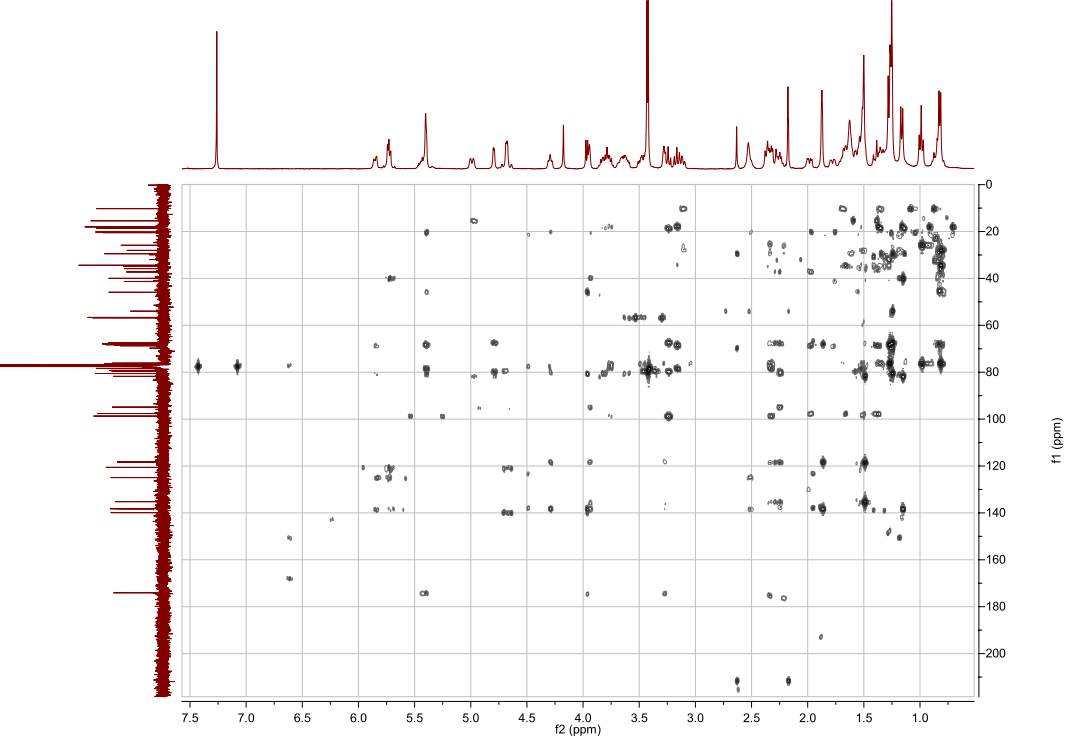

Supplement: Supplementary file 10 — Additional file 10: Figure S8. HMBC spectrum of compound 1. [file 12934_2015_337_MOESM10_ESM.docx]

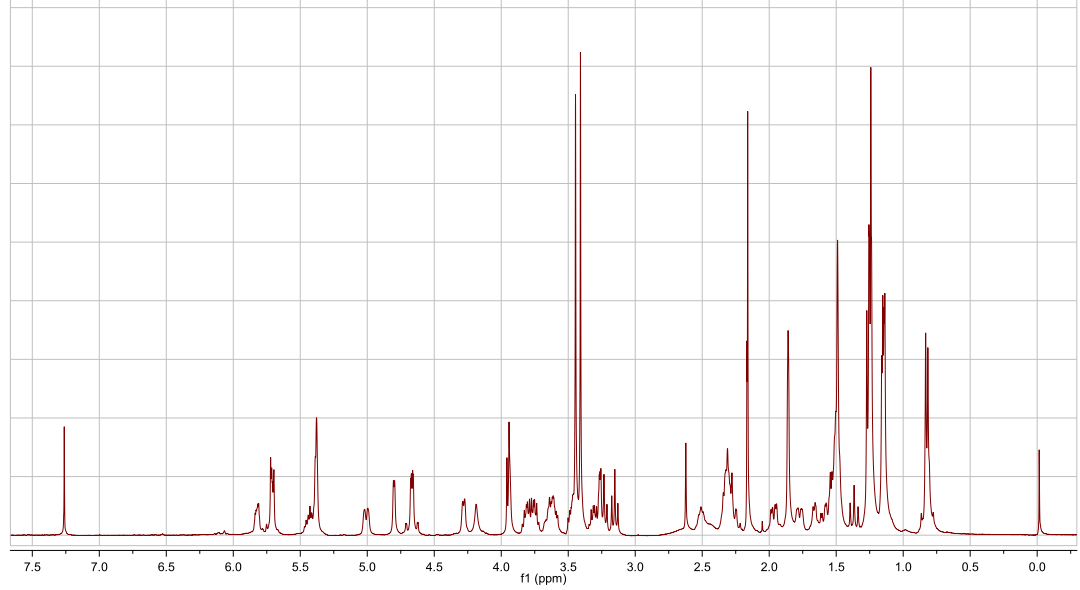

Supplement: Supplementary file 11 — Additional file 11: Figure S9. 1H NMR spectrum of compound 2 in CDCl3 (400 MHz). [file 12934_2015_337_MOESM11_ESM.docx]

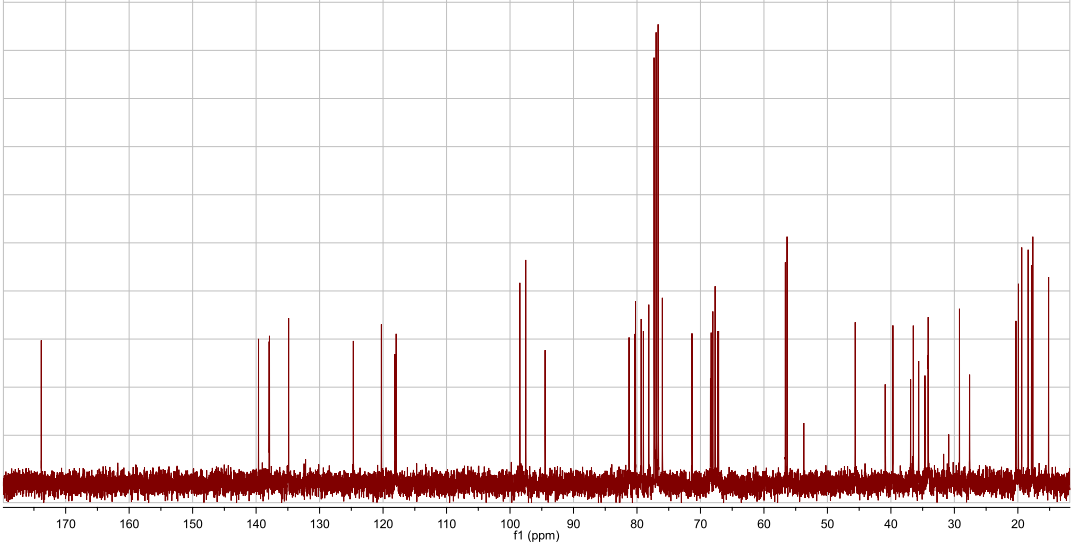

Supplement: Supplementary file 12 — Additional file 12: Figure S10. 13C NMR spectrum of compound 2 in CDCl3 (100 MHz). [file 12934_2015_337_MOESM12_ESM.docx]

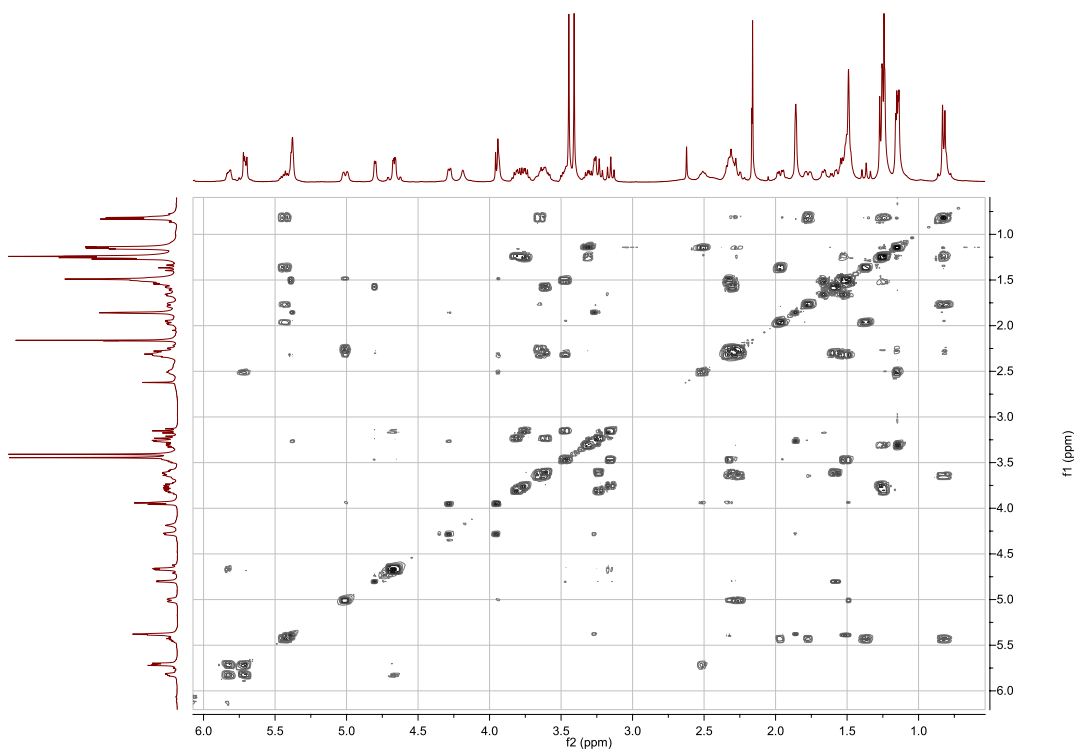

Supplement: Supplementary file 13 — Additional file 13: Figure S11. COSY spectrum of compound 2. [file 12934_2015_337_MOESM13_ESM.docx]

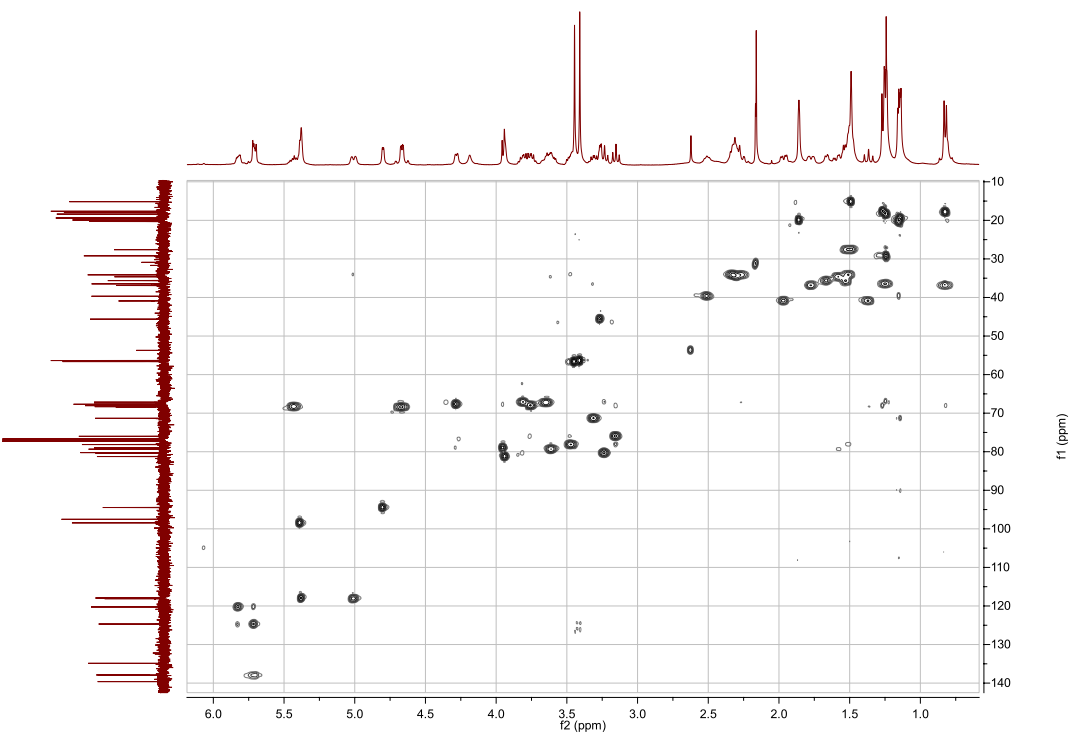

Supplement: Supplementary file 14 — Additional file 14: Figure S12. HSQC spectrum of compound 2. [file 12934_2015_337_MOESM14_ESM.docx]

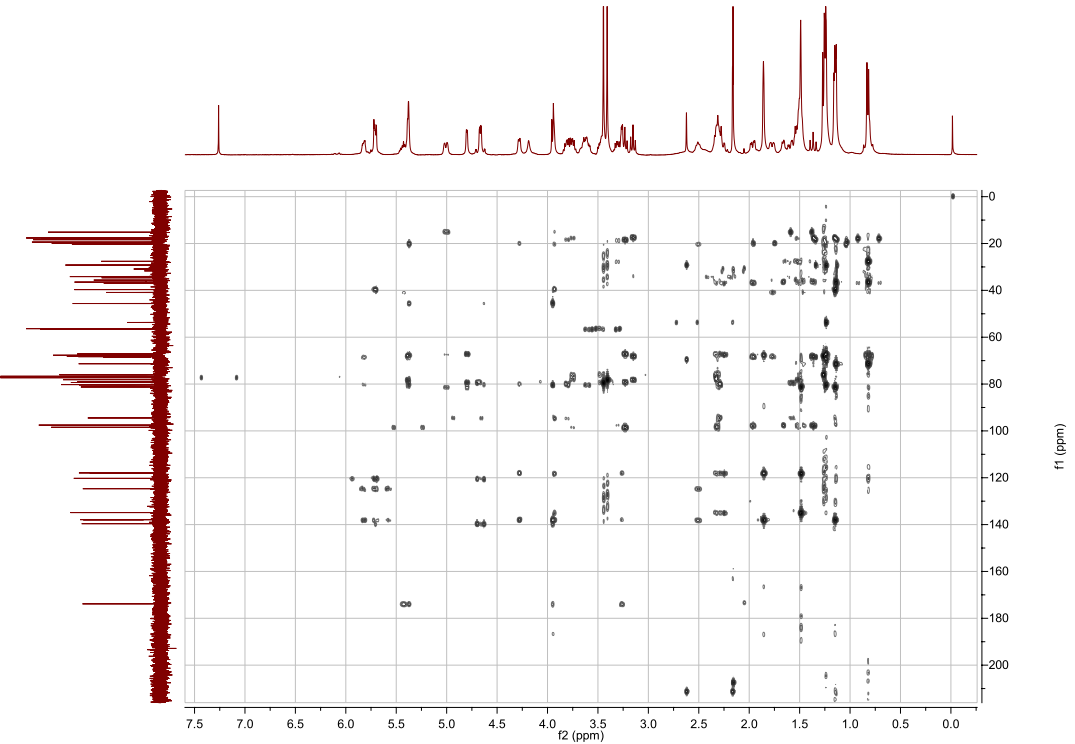

Supplement: Supplementary file 15 — Additional file 15: Figure S13. HMBC spectrum of compound 2. [file 12934_2015_337_MOESM15_ESM.docx]
