# Supplementary material for: Designed biosynthesis of 25-methyl and 25-ethyl ivermectin with enhanced insecticidal activity by domain swap of avermectin polyketide synthase
Source: Microb Cell Fact. 2015 Sep 24;14:152. doi: 10.1186/s12934-015-0337-y (PMC4581413; doi:10.1186/s12934-015-0337-y)
Supplement: Supplementary file 2 — Additional file 2: Table S1. Primers used for gene cloning, constructing and confirming the mutants. [file 12934_2015_337_MOESM2_ESM.docx]

| Primers | Sequences (5’→ 3’)^a^ |
| --- | --- |
| a1 | CCAAGCTTCCGCATTCATCTGCTCCG |
| a2 | GCTCTAGACCGGCTGCTGACACGTTGC |
| b1 | GCTCTAGACGCGGCGGAGCACCC |
| b2 | GCTCTAGATGCCCGGTCATGCCGCTGG |
| c1 | GCTCTAGACGGCACCGACCACCGG |
| c2 | CGGAATTCCTGCTCCAGGTTCCATCCAC |
| A1 | CCCAAGCTTCGAGCAACTGGGACGTG |
| A2 | GGGAAGACGAACGCGACGTCGGCCACTCCGAGGA |
| B1 | TCCTCGGAGTGGCCGACGTCGCGTTCGTCTTCCC |
| B2 | TCCTCGTCGAGGTGGCCCCGGGAGTGCAGATGG |
| C1 | ACCATCTGCACTCCCGGGGCCACCTCGACGAGGA |
| C2 | TGCTCTAGACACATCCACATCCGCCAC |
| E1 | GGACGACGGTACATGGACACG |
| E2 | TCGGCGTGGCGGGTGAGGGT |
| V1 | ATCTGCTGACGGTCTCCC |
| V2 | CCGGGAGTGCAGATGGTC |

^a^Underlined sequence for restriction enzyme recognition sites
