# Supplementary material for: Designed biosynthesis of 25-methyl and 25-ethyl ivermectin with enhanced insecticidal activity by domain swap of avermectin polyketide synthase
Source: Microb Cell Fact. 2015 Sep 24;14:152. doi: 10.1186/s12934-015-0337-y (PMC4581413; doi:10.1186/s12934-015-0337-y)
Supplement: Supplementary file 4 — Additional file 4: Table S2. 1H and 13C NMR data of compounds 1 and 2. [file 12934_2015_337_MOESM4_ESM.docx]

| Carbon | Compound **1** | | Compound **2** | |
| --- | --- | --- | --- | --- |
|  | *δ*_C_, type | *δ*_H_ (m, *J* in Hz) | *δ*_C_, type | *δ*_H_ (m, *J* in Hz) |
| 1 | 174.07, qC |  | 173.84, qC |  |
| 2 | 45.90, CH | 3.28 (m) | 45.62, CH | 3.27 (m) |
| 3 | 118.24, CH | 5.39 (m) | 117.93, CH | 5.38 (m) |
| 4 | 138.18, qC |  | 137.89, qC |  |
| 5 | 67.95, CH | 4.29 (d, *J*=6.3) | 67.69, CH | 4.28 (d, *J*=5.8) |
| 6 | 79.21, CH | 3.97 (d, *J*=6.2) | 78.99, CH | 3.96 (m) |
| 7 | 80.55, qC |  | 80.23, qC |  |
| 8 | 139.89, qC |  | 139.62, qC |  |
| 9 | 120.58, CH | 5.84 (br d, *J*=9.9) | 120.26, CH | 5.82 (m) |
| 10 | 124.94, CH | 5.72 (overlap) | 124.69, CH | 5.72 (m) |
| 11 | 138.26, CH | 5.73 (overlap) | 137.95, CH | 5.71 (m) |
| 12 | 39.96, CH | 2.51 (m) | 39.68, CH | 2.51 (m) |
| 13 | 81.76, CH | 3.94 (m) | 81.25, CH | 3.94 (m) |
| 14 | 135.21, qC |  | 134.87, qC |  |
| 15 | 118.44, CH | 4.99 (d, *J*=9.8) | 118.12, CH | 5.01 (d, *J*=9.4) |
| 16 | 34.42, CH_2_ | 2.28 (m) | 34.13, CH_2_ | 2.28 (m) |
| 17 | 67.52, CH | 3.65 (m) | 67.29, CH | 3.65 (m) |
| 18 | 37.24, CH_2_ | 0.83 (m) | 36.89, CH_2_ | 0.82 (m) |
|  |  | 1.77(br d, J=9.5) |  | 1.77(br d, *J*=9.5) |
| 19 | 68.61, CH | 5.43 (m) | 68.32, CH | 5.43 (m) |
| 20 | 41.31, CH_2_ | 1.38 (m) | 40.90, CH2 | 1.36 (t, J=11.8) |
|  |  | 1.98(dd, J=12.2, 4.3) |  | 1.97(dd, *J*=12.1, 4.3) |
| 21 | 97.63, qC |  | 97.52, qC |  |
| 22 | 35.82, CH_2_ | 1.52 (m) | 35.62, CH*2* | 1.52 (m) |
|  |  | 1.66 (br d, *J*=6.3, 1H) |  | 1.66 (br d, *J*=6.0) |
| 23 | 28.04, CH_2_ | 1.51(m) | 27.61, CH_2_ | 1.50 (m) |
| 24 | 34.39, CH | 1.32 (m) | 36.47, CH | 1.25 (m) |
| 25 | 76.14, CH | 3.12 (m) | 71.33, CH | 3.30 (dd, *J*=9.7, 6.3) |
| 26 | 25.86, CH_2_ | 1.35 (m) | 19.39, CH3 | 1.14 (d, J=6.0) |
|  |  | 1.68 (m) |  |  |
| 27 | 10.24, CH_3_ | 0.99 (t, *J*=7.2) |  |  |
| 4-Me | 20.21, CH_3_ | 1.87 (s) | 19.91, CH_3_ | 1.86 (s) |
| 8α | 68.72, CH_2_ | 4.65 (dd, *J*=14.6, 1.8) | 68.42, CH_2_ | 4.64 (dd, *J*=14.4, 1.9) |
|  |  | 4.70 (dd, *J*=14.6, 2.2) |  | 4.69 (dd, *J*=14.4, 1.9) |
| 12-Me | 20.48, CH_3_ | 1.15 (d, *J*=6.9) | 20.30, CH_3_ | 1.15 (d, *J*=6.9) |
| 14-Me | 15.42, CH_3_ | 1.50 (s) | 15.16, CH_3_ | 1.49 (s) |
| 24-Me | 17.99, CH_3_ | 0.82 (d, *J*=6.3) | 17.85, CH_3_ | 0.82 (d, *J*=6.6) |
| 1′ | 94.88, qC | 4.79 (d, *J*=3.1) | 94.47, qC | 4.80 (d, *J*=3.0) |
| 2′-a | 34.84, CH_2_ | 1.57 (m) | 34.63, CH_2_ | 1.58 (dd, *J*=12.3, 3.4) |
| 2′-b |  | 2.26 (m) |  | 2.30 (m) |
| 3′ | 79.57, CH | 3.62 (m) | 79.34, CH | 3.61 (m) |
| 4′ | 80.57, CH | 3.24 (t, *J*=9.1) | 80.35, CH | 3.23 (t, *J*=8.9) |
| 5′ | 67.42, CH | 3.81 (m) | 67.14, CH | 3.81 (m) |
| 6′ | 18.64, CH_3_ | 1.25 (d, *J*=6.1) | 18.38, CH_3_ | 1.24 (d, *J*=6.1) |
| 1′′ | 98.71, CH | 5.40 (d, *J*=3.7) | 98.45, CH | 5.39 (d, *J*=3.7) |
| 2′′-a | 34.36, CH_2_ | 1.51 (m) | 34.15, CH_2_ | 1.51 (m) |
| 2′′-b |  | 2.34 (m) |  | 2.32 (m) |
| 3′′ | 78.37, CH | 3.47 (m) | 78.14, CH | 3.47 (m) |
| 4′′ | 76.30, CH | 3.16 (t, *J*=9.1) | 76.00, CH | 3.15 (t, *J*=9.1) |
| 5′′ | 68.30, CH | 3.75 (m) | 68.05, CH | 3.75 (m) |
| 6′′ | 17.91, CH_3_ | 1.27 (d, *J*=6.4) | 17.64, CH_3_ | 1.26 (d, *J*=6.6) |
| 3′′-O-Me | 56.81, CH_3_ | 3.43 (s) | 56.63, CH_3_ | 3.45 (s) |
| 3′′-O-Me | 56.63, CH_3_ | 3.42 (s) | 56.36, CH_3_ | 3.41 (s) |
